# Cardiovascular risk factors among people with drug-resistant tuberculosis in Uganda

**DOI:** 10.1186/s12872-022-02889-y

**Published:** 2022-11-04

**Authors:** Joseph Baruch Baluku, Martin Nabwana, Joanitah Nalunjogi, Winters Muttamba, Ivan Mubangizi, Lydia Nakiyingi, Willy Ssengooba, Ronald Olum, Felix Bongomin, Irene Andia-Biraro, William Worodria

**Affiliations:** 1 Division of Pulmonology, Kiruddu National Referral Hospital, Kampala, Uganda; 2 Makerere University Lung Institute, Kampala, Uganda; 3 Directorate of programs, Mildmay Uganda, Wakiso, Uganda; 4 Makerere University-John Hopkin’s University Collaboration, Kampala, Uganda; 5 Department of Internal Medicine, School of Medicine, Makerere University College of Health Sciences, Kampala, Uganda; 6 Department of Internal Medicine, St. Francis Hospital, Nsambya, Kampala Uganda; 7 Department of Medical Microbiology and Immunology, Faculty of Medicine, Gulu University, Gulu, Uganda; 8 Department of Internal Medicine, le mémorial Hospital, Kampala, Uganda; 9 Division of Infection and Global Health, School of Medicine, University of St Andrews, St Andrews, UK; 10 Department of Medical Microbiology, Makerere University, Kampala, Uganda

**Keywords:** Smoking, Diabetes, Hypertension, Lipids, TB, Cardiovascular, Cholesterol, Obesity, MDR TB

## Abstract

**Background:**

Tuberculosis (TB) and its risk factors are independently associated with cardiovascular disease (CVD). We determined the prevalence and associations of CVD risk factors among people with drug-resistant tuberculosis (DRTB) in Uganda.

**Methods:**

In this cross-sectional study, we enrolled people with microbiologically confirmed DRTB at four treatment sites in Uganda between July to December 2021. The studied CVD risk factors were any history of cigarette smoking, diabetes mellitus (DM) hypertension, high body mass index (BMI), central obesity and dyslipidaemia. We used modified Poisson regression models with robust standard errors to determine factors independently associated with each of dyslipidaemia, hypertension, and central obesity.

**Results:**

Among 212 participants, 118 (55.7%) had HIV. Overall, 196 (92.5%, 95% confidence interval (CI) 88.0-95.3) had ≥ 1 CVD risk factor. The prevalence; 95% CI of individual CVD risk factors was: dyslipidaemia (62.5%; 55.4–69.1), hypertension (40.6%; 33.8–47.9), central obesity (39.3%; 32.9–46.1), smoking (36.3%; 30.1–43.1), high BMI (8.0%; 5.0–12.8) and DM (6.5%; 3.7–11.1). Dyslipidaemia was associated with an increase in glycated haemoglobin (adjusted prevalence ratio (aPR) 1.14, 95%CI 1.06–1.22). Hypertension was associated with rural residence (aPR 1.89, 95% CI 1.14–3.14) and previous history of smoking (aPR 0.46, 95% CI 0.21–0.98). Central obesity was associated with increasing age (aPR 1.02, 95%CI 1.00–1.03), and elevated diastolic blood pressure (aPR 1.03 95%CI 1.00–1.06).

**Conclusion:**

There is a high prevalence of CVD risk factors among people with DRTB in Uganda, of which dyslipidaemia is the commonest. We recommend integrated services for identification and management of CVD risk factors in DRTB.

## Background

Africa is undergoing rapid urbanisation which is associated with lifestyles that increase the risk of cardiovascular disease (CVD) [1]. As such, there is an increase in CVD risk factors and consequently CVDs across Africa. Most African countries are unlikely to achieve tobacco control targets by 2025 and a rapid increase in smoking prevalence is projected to occur among men by 2025 [2]. Additionally, an increase in the mean blood pressures was observed between 1975 and 2015 [3]. Further, Africa has seen an explosive increase in the prevalence of high body mass indices. Between 1990 and 2015, there was a 330% increase in the prevalence of “overweight” in Southern Africa, 73% rise in Northern Africa, 70% in West Africa and 9% in East Africa [4]. Lastly, there was a rise in the burden of diabetes mellitus (DM) between 1980 and 2014 from 3.4 to 8.5% in men and 4.1–8.9% in women in Africa [5]. A modelling study projects an exponential increase in DM in Africa between 2017 and 2025 [6]. A recent systematic review reported a high prevalence of several CVD risk factors in sub-Saharan Africa (SSA): hypertension (30%), hyperlipidaemia (25%), physical inactivity (22%), obesity (up to 40% in women and 15% in men), smoking (10%), and DM (3.5%) [7].

At the same time, Africa contributes a quarter of the global tuberculosis (TB) cases [8]. A convergence of CVD and TB is evident in the region [9, 10]. TB independently increases the risk for ischemic stroke [11], acute coronary syndrome [12] and myocardial infarction [13]. Therefore, a high prevalence of CVD among people with TB could synergistically increase the risk of death from major adverse cardiovascular events in these people [14]. However, the prevalence of CVD risk factors is not well established particularly in drug resistant TB (DRTB); which already posts a high mortality rate of 21% in Africa [15]. A study from South Africa reported that 52% of people with DRTB had at least one CVD risk factor, of whom 23% had moderate or severe CVD risk [16]. There is need for more studies to characterise CVD risk factors in DRTB in Africa to inform the need for integrating CVD prevention interventions in TB programs.

Uganda is among the high-burden TB and TB/HIV countries which has enrolled almost 2000 people with DRTB care since the inception of programmatic management of DRTB in 2013 [17–19]. The incidence of rifampicin resistant TB has increased by 20% between 2014 and 2018 [20]. About 10% of premature deaths in Uganda are attributed to CVD and 56% of adults are estimated to have at least two CVD risk factors [21, 22]. In this study, we determined the prevalence of CVD risk factors, and factors associated with selected CVD risk factors among people with DRTB in Uganda.

## Methods

### Study design, population, and setting

This was a cross sectional study at four DRTB treatment centres in Uganda. We purposively selected a high volume DRTB treatment site from each of the four regions of Uganda due to the geographical variation of hypertension and DM in the country [23, 24]. Between July to December 2021, participants were enrolled from Mulago, Mbarara, Lira, and Mbale referral hospitals in central, western, northern, and eastern Uganda, respectively. Eligible participants were adults (age ≥ 18 years) with bacteriologically confirmed TB, and any form of drug resistance who were receiving treatment at these sites during the period of data collection. We consecutively enrolled participants during the clinic appointment days at the study sites. The programmatic management of DRTB in Uganda has been extensively described recently [19].

### Data collection and study measurements

Trained research assistants administered a pretested questionnaire to eligible participants for socio-demographic characteristics, medical history, any history of cigarette smoking and alcohol use. Participants underwent anthropometric measurements of their weight, height and waist/hip ratio using a weighing scale (Seca 760®), stadiometer (Seca 213®) and tape measure respectively. The body mass index (BMI) was calculated using the formula; BMI = weight (kilograms)/height (in metres)^2^. Using a battery powered digital blood pressure (BP) machine (Omron®, Hem 7120), the BP was taken on two separate occasions, 20 min apart, at the DRTB treatment centre. The average BP of the two measurements was considered as the participant’s BP. A study nurse drew 4 millilitres (mls) of blood which was tested for random blood glucose (RBG), glycated haemoglobin (HbA1c), non-fasting lipid profile, and a complete blood count. The RBG was measured using a point of care glucometer (Accu-Chek®). The HbA1c and blood lipids (triglycerides, total cholesterol, low density lipoprotein cholesterol (LDL-c), and high-density lipoprotein cholesterol (HDL-c)) were estimated using the Cobas® 6000 analyzer series (Roche Diagnostics, USA). HIV infection was confirmed using immunochromatographic tests according to the Uganda HIV testing guidelines [25].

### Study outcomes

The main study outcome was the prevalence of CVD risk factors. The CVD risk factors of focus were any history of cigarette smoking, DM (HbA1c ≥ 6.5% and/or RBS ≥ 11.1 mmol/l with symptoms and/or use of anti-hyperglycaemic agents) [26], Hypertension (systolic ≥ 140 mmHg and/or diastolic ≥ 90 mmHg and/or use of anti-hypertension medication) [27], central obesity (waist circumference of ≥ 102/88 cm and/or a waist-hip ratio of ≥ 0.90/0.85 in males/females) [27], high BMI (BMI ≥ 25 kg/meters^2^), and dyslipidaemia (total cholesterol of > 5.0 mmol/l [28] and/or LDL-c of > 4.14 mmol/l [29], and/or triglyceride level of ≥ 1.7 mmol/l, and/or HDL-c of < 1.03 mmol/l for men and < 1.29 mmol/l for women [30]).

### Sample size estimation and statistical analysis

There were approximately 520 people receiving DRTB treatment in 2021. A sample size of 221 would be adequate to determine the prevalence of CVD risk factors, assuming a prevalence of 52.3% of any CVD risk factor and a 95% confidence interval (CI) [16, 31]. We, however, conducted a census of all people with DRTB at the study sites. Data were entered in EpiData 4.4.0 and exported to Stata 16.0 for analysis. Continuous variables were presented as medians with the corresponding interquartile ranges (IQR). Categorical data were presented as proportions. The prevalence of CVD risk factors was calculated as the proportion of people with a given risk factor to the total number of people with DRTB. The corresponding 95% confidence intervals (CI) were also estimated. We used modified Poisson regression models with robust standard errors to determine factors independently associated with each of dyslipidaemia, hypertension, and central obesity. The few counts for DM and high BMI precluded us from performing analyses for factors associated with these risk factors. For all models, we included age, sex, HIV status, residence, BP, blood lipids, HbA1c, alcohol use, smoking, BMI, waist-hip ratio, waist circumference except when the CVD risk factor is measured using one of these variables.

## Results

### Characteristics of study participants

We enrolled 212 participants of whom 156 (73.6%) were male and 118 (55.7%) had HIV co-infection. The median (IQR) age was 37 (30–46) years (n = 211). The median (IQR) BMI was 19.7 (17.7–22.2) Kg/m^2^ (n = 199) while the systolic and diastolic BPs were 124.0 (116.0–133.5) mmHg and 85.3 (76.5–93.0) mmHg, respectively. The median (IQR) RBG was 4.6 (4.0–5.7) mmol/l while the HbA1c was 4.7% (3.9–5.2). Regarding the blood lipids, the median (IQR) values were as follows: total cholesterol (3.4 [2.7–4.2] mmol/l), LDL-c (1.7 [1.2–2.2] mmol/l), HDL-c (1.2 [0.9–1.7] mmol/l) and triglycerides (1.2 [0.87–1.62] mmol/l). Overall, 77 (78.6%) reported a family history of CVD (including pre-mature CVD death). Table 1 shows the characteristics of the study participants.

**Table 1:** Characteristics of study participants

Characteristic	Frequency (n = 212)		Percentage		
DRTB treatment site					
Lira Regional Referral Hospital (Northern region)	85		40.1		
Mulago National Referral Hospital (Central region)	83		39.1		
Mbale Regional Referral Hospital (Eastern region)	25		11.8		
Mbarara Regional Referral Hospital (Western region)	19		9.0		
Rural residence	128		60.4		
Married	112		52.8		
Education (n = 211)					
None	18		8.5		
Primary-level education	122		57.8		
Secondary-level education	55		26.1		
Tertiary-level education	16		7.6		
Employment status					
Peasant farmer	92		43.4		
Self employed	48		22.6		
Unemployed	38		17.9		
Formal employment	34		16.0		
Family history of cardiovascular disease (n=98)					
Hypertension	55		56.1		
Diabetes mellitus	25		25.5		
Obesity (n = 97)	8		8.3		
Heart failure (n=97)	8		8.3		
Kidney disease	7		7.1		
Stroke	6		6.1		
Premature CVD death in first degree relative**	5		5.1		
Any history of alcohol use	148		69.8		
Type of DRTB at Baseline (n = 210)					
Rifampicin resistance/multidrug resistant	204		96.2		
Pre- XDRTB	3		1.43		
Poly resistant tuberculosis	1		0.5		
XDR-TB	1		0.5		
Mono-resistance	1		0.5		
Previous TB episode (n = 210)	112		53.3		
Mycobacterial load at treatment initiation* (n = 170)					
High	52		30.6		
Medium	35		20.6		
Low	28		16.5		
Very low	55		32.4		
Drugs in the treatment regimen (n = 209)					
Levofloxacin	200		95.7		
Cycloserine	197		94.3		
Clofazimine	197		94.3		
Linezolid	165		79.0		
Bedaquiline	157		75.1		
Pyrazinamide	39		18.7		
Ethionamide	10		4.8		
Delamanid	8		3.8		
Ethambutol	6		2.9		
Moxifloxacin	2		1.0		
Amikacin	1		0.5		
Time from diagnosis to treatment (days), median (IQR), n= 202	8 (5 – 14)				

*Determined by cycle threshold values of the MTB Xpert/Rif assay. ** (age <55 for male and <65 for female relatives). Abbreviations: DRTB – drug resistant tuberculosis, CVD – cardiovascular disease, XDRTB – extensively drug resistant tuberculosis (defined as resistance to rifampicin, isoniazid, a fluoroquinolone and an injectable aminoglycoside)

### Prevalence and distribution of CVD risk factors

Overall, 196 (92.5%, 95% CI: 88.0–95.3) participants had ≥ 1 CVD risk factor. The median (IQR) number of CVD risk factors was 2 (1–2) per participant. Overall, 64 (32.7%) had one CVD risk factor, 87 (44.4%) had two, 36 (18.4%) had three and 9 (4.6%) had ≥ 4. The prevalence; 95% CI of CVD risk factors was: dyslipidaemia (62.5%; 55.4–69.1), hypertension (40.6%; 33.8–47.9), central obesity (39.3%; 32.9–46.1), smoking (36.3%; 30.1–43.1), high BMI (8.0%; 5.0–12.8) and DM (6.5%; 3.7–11.1). The prevalence of the individual CVD risk factors is summarised in Table 2.

**Table 2:** The prevalence of the CVD risk factors in DRTB

CVD risk factor in DRTB	Number with CVD risk factor	Prevalence (95% confidence interval) (%)
Hypertension (n = 187)^a^	76	40.6 (33.8 – 47.9)
Central obesity (n = 211)	83	39.3 (32.9 – 46.1)
History of smoking (n = 212)**	77	36.3 (30.1 – 43.1)
Dyslipidaemia (n = 192)	120	62.5 (55.4 – 69.1)
High total cholesterol (n = 162) High LDL-c Low HDL-c High triglycerides (n = 190)	24	14.8 (10.1 – 21.2)
	25	13.0 (8.9 – 18.6)
	76	39.6 (32.9 – 46.7)
	41	21.6 (16.3 – 28.0)
High BMI* (n = 199)	16	8.0 (5.0 – 12.8)
Diabetes mellitus (n = 186)^b^	12	6.5 (3.7 – 11.1)

*8 (4.0%) had BMI of ≥ 30 kg/m^2^, **9 (4.3%) were current smokers, ^a^only 4 were known to have hypertension, ^b^only 3 had known diabetes, another 16 (8.7%) had HbA1c ≥5.6% (pre-diabetes). Abbreviations: LDL-c – low density lipoprotein cholesterol, HDL-c – high density lipoprotein cholesterol, CVD – cardiovascular disease, DRTB – drug resistant tuberculosis, BP – blood pressure, BMI – body mass index

### Prevalence of CVD risk factors in DRTB by region of Uganda

Table 3 shows the prevalence of CVD risk factors among people with DRTB by region in Uganda. Northern Uganda posted the highest prevalence of hypertension (49.0% [95% CI 36.7–61.8], p = 0.003), while Eastern Uganda had the highest prevalence of dyslipidaemia (92.0% [95% CI 71.5–98.1], p = 0.003). The prevalence of central obesity was highest in Western Uganda (63.2% [95% CI 38.7–82.3], p = 0.052), although the difference across the regions was not statistically significant.

**Table 3:** The prevalence of the CVD risk factors in DRTB

CVD risk factor in DRTB	Number with CVD risk factor (%)	p-value*	Prevalence (95% CI)
At least one CVD risk factor	**196 (100.0)**	**0.839**	**92.5 (88.0 – 95.3)**
Central	76 (38.8)		91.6 (83.2 – 96.0)
Eastern	24 (12.2)		96.0 (74.5 – 99.5)
Western	17 (8.7)		89.5 (63.9 -97.6)
Northern	79 (40.3)		92.9 (85.0 – 96.8)
Hypertension (n = 187)	**76 (100.0)**	**0.003**	**40.6 (33.8 – 47.9)**
Central	39 (51.3)		47.0 (36.4 – 57.9)
Eastern	4 (5.3)		16.0 (63.0 – 94.2)
Western	3 (4.0)		16.7 (5.0 – 43.2)
Northern	30 (39.5)		49.0 (36.7 – 61.8)
Central obesity (n = 211)	**83 (100.0)**	**0.052**	**39.3 (32.9 – 46.1)**
Central	35 (42.2)		42.2 (31.9 – 53.2)
Eastern	6 (7.2)		24.0 (10.7 – 45.4)
Western	12 (14.5)		63.2 (38.7 – 82.3)
Northern	30 (36.1)		35.7 (26.1 – 46.6)
History of smoking (n = 212)	**77 (100.0)**	**0.145**	**36.3 (30.1 – 43.1)**
Central	26 (33.8)		31.3 (22.2 – 42.2)
Eastern	9 (11.7)		36.0 (19.2 – 57.1)
Western	4 (5.2)		21.1 (7.6 – 46.5)
Northern	38 (49.4)		44.7 (34.4 – 55.5)
Dyslipidaemia (n = 192)	**120 (100.0)**	**0.003**	**62.5 (55.4 – 69.1)**
Central	39 (32.5)		51.3 (40.0 – 62.5)
Eastern	23 (19.2)		92.0 (71.5 – 98.1)
Western	11 (9.2)		57.9 (34.1 – 78.5)
Northern	47 (39.2)		65.3 (53.4 – 75.5)
High BMI (n = 199)	**16 (100.0**)	**0.588**	**8.0 (5.0 – 12.8)**
Central	8 (50.0)		9.8 (4.9 – 18.5)
Eastern	3 (18.8)		12.0 (3.7 – 32.7)
Western	1 (6.3)		6.3 (0.7 – 37.6)
Northern	4 (25.0)		5.3 (2.0 – 13.4)
Diabetes mellitus (n = 186)	**12 (100.0)**	**0.150**	**6.5 (3.7 – 11.1)**
Central	2 (16.7)		2.5 (0.6 – 9.5)
Eastern	2 (16.7)		8.0 (1.9 – 28.5)
Western	1 (8.3)		5.9 (0.7 – 35.7)
Northern	7 (58.3)		11.1 (5.3 – 21.8)

*p-value compares the prevalence by regions, Abbreviations: BMI – body mass index, CI – confidence interval, CVD – cardiovascular disease

### Factors associated with CVD risk factors among people with DRTB in Uganda

Tables 4, 5 and 6 show factors associated with individual CVD risk factors. Dyslipidaemia was associated with an increase in glycated haemoglobin (adjusted prevalence ratio (aPR) 1.14, 95%CI 1.06–1.22, p = 0.001). Hypertension was associated with rural residence (aPR 1.89, 95% CI 1.14–3.14, p = 0.014) and previous history of smoking (aPR 0.46, 95% CI 0.21–0.98, p = 0.045). Central obesity was associated with increasing age (aPR 1.02, 95%CI 1.00–1.03, p = 0.040), and elevated diastolic BP (aPR 1.03, 95%CI 1.00–1.06, p = 0.043).

**Table 4:** Factors associated with hypertension among people with DRTB in Uganda

Variable	Crude prevalence ratio (PR) (95%CI)	p-value	Adjusted PR (95%CI)	p-value
Age	1.00 (0.99 – 1.02)	0.718	1.01 (0.99 – 1.04)	0.197
Sex				
Male	Reference		Reference	
Female	1.10 (0.76 – 1.59)	0.626	0.85 (0.51 – 1.43)	0.550
Residence				
Urban	Reference		Reference	
Rural	1.39 (0.96 – 2.01)	0.084	1.89 (1.14 – 3.14)	0.014
Alcohol use				
Never used alcohol	Reference		Reference	
Former user (≤6 months)	0.84 (0.57 – 1.22)	0.356	1.19 (0.73 – 1.93)	0.485
Current user (>6 months)	0.70 (0.43 – 1.13)	0.147	0.86 (0.44 – 1.66)	0.647
Smoking history				
Never smoked	Reference		Reference	
Former smoker (≤6 months)	0.62 (0.40 – 0.97)	0.036	0.46 (0.21 – 0.98)	0.045
Current smoker (>6 months)	0.53 (0.16 – 1.79)	0.308	0.54 (0.10 – 2.96)	0.477
HIV status				
Positive	Reference		Reference	
Negative	0.84 (0.58 – 1.20)	0.325	1.29 (0.76 – 2.18)	0.338
Total cholesterol	0.98 (0.87 – 1.10)	0.701	1.06 (0.84 – 1.34)	0.607
LDL-c	0.99 (0.86 – 1.13)	0.839	0.79 (0.56 – 1.12)	0.183
HDL-c	0.90 (0.68 – 1.20)	0.469	0.91 (0.52 – 1.57)	0.732
Triglycerides	1.14 (0.89 – 1.45)	0.296	0.93 (0.69 – 1.27)	0.661
Body mass index	0.99 (0.96 – 1.03)	0.736	0.99 (0.95 – 1.04)	0.743
Glycated haemoglobin	0.99 (0.82 – 1.19)	0.876	0.73 (0.51 – 1.05)	0.089
Random blood sugar	1.03 (0.95 – 1.12)	0.427	1.04 (0.96 – 1.13)	0.342
Waist-Hip ratio	0.71 (0.10 – 5.06)	0.731	0.08 (0.002 – 2.25)	0.138
Waist Circumference	1.01 (0.99 – 1.02)	0.295	1.03 (1.00 – 1.06)	0.061

Abbreviations: LDL-c – low density lipoprotein cholesterol, HDL-c – high density lipoprotein cholesterol

**Table 5:** Factors associated with dyslipidaemia among people with DRTB in Uganda

Variable	Crude prevalence ratio (PR) (95%CI)	p-value	Adjusted PR (95%CI)	p-value
Age	1.00 (0.99 – 1.01)	0.841	1.00 (0.99 – 1.01)	0.921
Sex				
Male	Reference		Reference	
Female	1.27 (1.02 – 1.57)	0.032	1.14 (0.86 – 1.51)	0.361
Residence				
Urban	Reference		Reference	
Rural	1.12 (0.89 – 1.42)	0.318	1.13 (0.87 – 1.46)	0.355
Alcohol use				
Never used alcohol	Reference		Reference	
Former user (≤6 months)	0.85 (0.68 – 1.08)	0.181	0.96 (0.73 – 1.26)	0.757
Current user (>6 months)	0.74 (0.54 – 1.01)	0.060	0.88 (0.59 – 1.30)	0.519
Smoking history				
Never smoked	Reference		Reference	
Former smoker (≤6 months)	0.79 (0.60 – 1.03)	0.078	0.95 (0.68 – 1.32)	0.769
Current smoker (>6 months)	1.17 (0.80 – 1.69)	0.417	1.41 (0.69 – 2.93)	0.346
HIV status				
Positive	Reference		Reference	
Negative	0.98 (0.78 – 1.220	0.823	0.95 (0.75 – 1.20)	0.643
Body mass index	0.99 (0.97 – 1.02)	0.497	0.99 (0.96 – 1.02)	0.412
Glycated haemoglobin	1.10 (1.00 – 1.20)	0.052	1.14 (1.06 – 1.22)	0.001
Random blood sugar	1.02 (0.99 – 1.05)	0.123	1.02 (0.98 – 1.06)	0.332
Waist-Hip ratio	0.43 (0.21 – 0.88)	0.021	0.30 (0.07 – 1.29)	0.106
Waist circumference	1.00 (0.99 – 1.01)	0.806	1.01 (0.99 – 1.02)	0.368
Systolic blood pressure	0.99 (0.98 – 1.00)	0.072	1.00 (0.98 – 1.01)	0.490
Diastolic blood pressure	0.99 (0.99 – 1.00)	0.207	1.00 (0.98 – 1.01)	0.855

**Table 6:** Factors associated with central obesity among people with DRTB in Uganda

Variable	Crude prevalence ratio (PR) (95%CI)	p-value	Adjusted PR (95%CI)	p-value
Age	1.02 (1.01 – 1.03)	<0.001	1.02 (1.00 – 1.03)	0.040
Sex				
Male	Reference		Reference	
Female	1.06 (0.73 – 1.54)	0.755	1.54 (0.95 – 2.51)	0.079
Residence				
Urban	Reference		Reference	
Rural	0.95 (0.68 – 1.34)	0.738	0.94 (0.63 – 1.42)	0.786
Alcohol use				
Never used alcohol	Reference		Reference	
Former user (≤6 months)	1.17 (0.79 – 1.73)	0.428	0.89 (0.54 – 1.47)	0.625
Current user (>6 months)	0.87 (0.52 – 1.45)	0.597	0.83 (0.44 – 1.57)	0.573
Smoking history				
Never smoked	Reference		Reference	
Former smoker (≤6 months)	1.16 (0.82 – 1.64)	0.401	1.34 (0.79 – 2.26)	0.278
Current smoker (>6 months)	0.58 (0.17 – 2.03)	0.397	0.91 (0.25 – 3.33)	0.888
HIV status				
Positive	Reference		Reference	
Negative	0.95 (0.68 – 1.34)	0.738	1.00 (0.67 – 1.50)	0.997
Total cholesterol	0.91 (0.80 – 1.03)	0.122	0.89 (0.75 – 1.05)	0.169
LDL-c	0.90 (0.77 – 1.05)	0.186	0.85 (0.69 – 1.04)	0.120
HDL-c	0.87 (0.67 – 1.13)	0.308	1.18 (0.78 – 1.80)	0.425
Triglycerides	1.23 (0.997 – 1.51)	0.053	1.08 (0.81 – 1.44)	0.591
Body mass index	1.02 (0.995 – 1.05)	0.108	1.02 (1.00 – 1.05)	0.234
Glycated haemoglobin	0.99 (0.87 – 1.12)	0.841	0.99 (0.81 – 1.22)	0.957
Random blood sugar	1.06 (1.02 – 1.10)	0.001	1.04 (0.99 – 1.09)	0.112
Systolic blood pressure	1.00 (0.99 – 1.02)	0.407	0.98 (0.96 – 1.01)	0.167
Diastolic blood pressure	1.01 (0.998 – 1.02)	0.096	1.03 (1.00 – 1.06)	0.043

Abbreviations: LDL-c – low density lipoprotein cholesterol, HDL-c – high density lipoprotein cholesterol

## Discussion

People with TB have a 51% higher risk of major adverse cardiovascular events [14]. In this multi-center study in Uganda, we determined the prevalence of CVD risk factors and associations of the individual risk factors among people with DRTB. We found that 9 in 10 people with DRTB have at least one CVD risk factor. Dyslipidaemia was the most prevalent (> 60%) while hypertension and central obesity were observed in approximately 40% of people. Smoking history was found in more than one-third of the participants. High BMI and DM were the least prevalent and were found in < 10%. The prevalence of the CVD risk factors in our study is higher than what is reported in the general population for hypertension (26.4%) [32], central obesity (11.8%) [33], cigarette smoking (9%) [34], and DM (1.4%). However it is lower than what is reported in the general population for high BMI (22.4%) [32] and dyslipidaemia (32–71%) [35, 36]. A high prevalence of CVD risk factors among people with TB is concerning because of their potential to worsen TB treatment outcomes and all-cause mortality even after TB cure [37]. Our findings, therefore, call for the integration of CVD risk reduction strategies in TB care in Uganda and similar TB high-burden settings. Certain regions posted a higher prevalence of CVD than others. That is, dyslipidaemia was highest in Eastern Uganda, smoking was highest in northern Uganda while high BMI was highest in Western Uganda. This calls for region-specific interventions to reduce CVD among people with DRTB in these settings.

Hypertension in TB has received little attention in literature. However, the prevalence of hypertension is on the rise in SSA [3]. A recent study from Guinea-Bissau has shown that hypertension is associated with poor TB treatment outcomes and a 64% higher risk of death after TB cure [38]. Therefore, BP control is likely to improve TB outcomes and reduce post-TB mortality. This is particularly important in Uganda where only 8% of people with hypertension are aware of their condition [32]. Similar to our findings, a review by Seegert and colleagues reported the prevalence of hypertension of up to 38.3% among people with TB (without DM) [39]. It is unclear why we observed a high prevalence of hypertension. However, inflammation due to chronic infection increases expression of endothelial adhesion molecules, oxidative stress, immune cell activation and infiltration in the kidney and blood vessels that result in vascular resistance and kidney remodelling [40].

Although smoking is an established risk factor for over 30 CVDs, smoking cessation programs are not integrated in TB care [41, 42]. At the same time, smoking adversely affects TB outcomes [43]. Short health workers’ training can increase confidence of health workers to elicit smoking history from people with TB and offer smoking cessation support [44]. This might result in BP reduction as suggested by the association between smoking cessation and a lower risk of hypertension in our study. The high prevalence of smoking in TB is likely because smoking increases one’s risk for TB. Nicotine in tobacco attenuates innate immune responses against TB by decreasing expression of toll-like receptors and production of cytokines (IL – 6 and 8 and TNFα) and chemokines by lung epithelial cells, macrophages and type 2 pneumocytes [45, 46]. Kirenga and colleagues reported a lower prevalence of smoking of 26% among people with drug sensitive TB in Uganda compared to our study [47]. In comparison, Whitehouse and colleagues reported a prevalence of 31% in DRTB, similar to our study [16]. It is likely that people with DRTB have higher smoking rates than drug sensitive TB [48].

It was interesting to observe a disconnect in the prevalence of obesity as measured by the BMI (4.0%) and waist-hip ratio/waist circumference (39.3%) in our study. In fact, undernutrition, as measured by the BMI is very prevalent among people with DRTB in Uganda [49]. However, similar to our findings, central obesity was prevalent in 33% of people with TB and it correlated with DM better than the BMI in a study from the Philippines [50]. A similar disconnect was observed between the prevalence of obesity by the BMI (3.4%) and waist circumference (13.7%) in Bangladesh [51]. If follows that those measures of central obesity may be better predictors of CVD among people with TB than the BMI, as has been reported in the general population [52]. In support of this, our results showed an association between central obesity with hypertension and increasing age which are predictors of cardiovascular events. Conversely, the BMI was not associated with any of the other CVD risk factors.

Very few studies have evaluated lipid abnormalities among people with TB in Africa. Similar to our study, Lawson and colleagues found that 29% and 50% of people with TB had elevated LDL-c and low HDL-c, respectively, in Nigeria [53]. The effect of high lipids on CVD risk and TB outcomes in people with TB is unclear. However, high lipid levels might favour entry and survival of *Mycobacterium tuberculosis* in the macrophage [54]. Conversely, statins reduce the risk of active TB and this may be by lowering cholesterol; an essential molecule for internalisation of *M. tuberculosis* in host immune cells [55]. Randomised controlled trials are needed to determine the role of statins in CVD prevention in people with active TB and TB survivors. This might be particularly important among people with TB and DM since an association between HbA1c and dyslipidaemia was evident in our study.

The prevalence of DM observed in our study (6.5%) is similar to that reported among people with DRTB in South Africa (5.2%) [16] but lower than the estimated prevalence of DM in TB in Africa (9.0%) [56]. Higher estimates of DM in TB could be due to transient hyperglycaemia that tends to resolve with TB treatment [57]. Our study population was well established on treatment (a median of 8 months into therapy). Our estimate is therefore likely to be reliable. Screening for DM among people with DRTB should be prioritised because DM is a known risk factor for DRTB and predicts TB treatment failure, relapse and death [58, 59].

Our study has some limitations. We did not evaluate the prevalence of physical inactivity among people with DRTB. However, exercise intolerance due to respiratory symptoms would affect assessing this CVD risk factor. We also did not evaluate for hazardous alcohol use to better characterise the effect of alcohol on other CVD risk factors. Nonetheless, estimating the prevalence of hazardous alcohol use is difficult in Africa. This is because the types of alcoholic drinks are varied (often home brewed) and difficult to quantify; while the most common pattern of alcohol consumption is heavy episodic use [60]. Similarly, we did not quantify cigarette smoking, by say the number of pack years. It is, however, important to note that former smokers and people who use as low as one cigarette a day have elevated CVD risk than never smokers [61, 62]. Therefore, using any history of smoking is justified. Lastly, we did not have a control group (people without TB) to compare the prevalence of CVD risk factors. We, however, have compared our findings with the reported prevalence in the general Ugandan population.

## Conclusion

We found a very high prevalence of CVD risk factors among people with DRTB. Dyslipidaemia, hypertension, and smoking were the most prevalent. The prevalence of DM and central obesity were also higher than the estimates in the Ugandan population. There is an urgent need to integrate cost-effective screening strategies for CVD risk factors in DRTB care.

## Data Availability

The datasets used and/or analysed during the current study are available from the corresponding author on reasonable request.

## Abbreviations

CVD: Cardiovascular disease
BP: Blood pressure
LDL-c: Low density lipoprotein cholesterol
HDL-c: High density lipoprotein cholesterol
RBG: Random blood glucose
DM: Diabetes mellitus
HbA1c: Glycated haemoglobin
BMI: Body mass index
TB: Tuberculosis
DRTB: Drug resistant tuberculosis
CI: Confidence interval
IQR: Interquartile range
PR: Prevalence ratio
